# Seaweed Essential Oils as a New Source of Bioactive Compounds for Cyanobacteria Growth Control: Innovative Ecological Biocontrol Approach

**DOI:** 10.3390/toxins12080527

**Published:** 2020-08-17

**Authors:** Soukaina El Amrani Zerrifi, Fatima El Khalloufi, Richard Mugani, Redouane El Mahdi, Ayoub Kasrati, Bouchra Soulaimani, Lillian Barros, Isabel C. F. R. Ferreira, Joana S. Amaral, Tiane Cristine Finimundy, Abdelaziz Abbad, Brahim Oudra, Alexandre Campos, Vitor Vasconcelos

**Affiliations:** 1 Water, Biodiversity and Climate Change Laboratory, Phycology, Biotechnology and Environmental Toxicology Research Unit, Faculty of Sciences Semlalia Marrakech, Cadi Ayyad University, P.O. Box 2390, 40000 Marrakech, Morocco; soukainaelamranizerrifi@gmail.com (S.E.A.Z.); richardmugani@gmail.com (R.M.); redouane.elmahdii@gmail.com (R.E.M.); oudra@uca.ac.ma (B.O.); 2 Laboratory of Chemistry, Modeling and Environmental Sciences, Polydisciplinary Faculty of Khouribga, Sultan Moulay Slimane University of Beni Mellal, P.B. 145, 25000 Khouribga, Morocco; elkhalloufi.f@gmail.com; 3 Department of Health and Agro-Industry Engineering, High School of Engineering and Innovation of Marrakesh (E2IM), Private University of Marrakesh (UPM), 42312 Marrakech, Morocco; ayoub.kasrati@gmail.com; 4 Laboratory of Microbial Biotechnologies, Agrosciences and Environment, Faculty of Science Semlalia Marrakech, Cadi Ayyad University, P.O. Box 2390, 40000 Marrakech, Morocco; bouchrasoulaimanigebc@gmail.com (B.S.); abbad.abdelaziz@gmail.com (A.A.); 5 Centro de Investigação de Montanha (CIMO), Instituto Politécnico de Bragança, Campus de Santa Apolónia, 5300-253 Bragança, Portugal; lillian@ipb.pt (L.B.); iferreira@ipb.pt (I.C.F.R.F.); jamaral@ipb.pt (J.S.A.); tcfinimu@hotmail.com (T.C.F.); 6 REQUIMTE-LAQV, Faculdade de Farmácia, Universidade do Porto, 4050-313 Porto, Portugal; 7 CIIMAR, Interdisciplinary Centre of Marine and Environmental Research, University of Porto, Terminal de Cruzeiros do Porto de Leixões, Av. General Norton de Matos, s/n, 4450-208 Matosinhos, Portugal; acampos@ciimar.up.pt; 8 Departament of Biology, Faculty of Sciences, University of Porto, Rua do Campo Alegre, 4169-007 Porto, Portugal

**Keywords:** anti-cyanobacterial activity, bio-control, seaweed essential oils, *Microcystis aeruginosa*

## Abstract

The application of natural compounds extracted from seaweeds is a promising eco-friendly alternative solution for harmful algae control in aquatic ecosystems. In the present study, the anti-cyanobacterial activity of three Moroccan marine macroalgae essential oils (EOs) was tested and evaluated on unicellular *Microcystis aeruginosa* cyanobacterium. Additionally, the possible anti-cyanobacterial response mechanisms were investigated by analyzing the antioxidant enzyme activities of *M. aeruginosa* cells. The results of EOs GC–MS analyses revealed a complex chemical composition, allowing the identification of 91 constituents. Palmitic acid, palmitoleic acid, and eicosapentaenoic acid were the most predominant compounds in *Cystoseira tamariscifolia, Sargassum muticum*, and *Ulva lactuca* EOs, respectively. The highest anti-cyanobacterial activity was recorded for *Cystoseira tamariscifolia* EO (ZI = 46.33 mm, MIC = 7.81 μg mL^−1^, and MBC = 15.62 μg mL^−1^). The growth, chlorophyll-*a* and protein content of the tested cyanobacteria were significantly reduced by *C. tamariscifolia* EO at both used concentrations (inhibition rate >67% during the 6 days test period in liquid media). Furthermore, oxidative stress caused by *C. tamariscifolia* EO on cyanobacterium cells showed an increase of the activities of superoxide dismutase (SOD) and catalase (CAT), and malondialdehyde (MDA) concentration was significantly elevated after 2 days of exposure. Overall, these experimental findings can open a promising new natural pathway based on the use of seaweed essential oils to the fight against potent toxic harmful cyanobacterial blooms (HCBs).

## 1. Introduction

On the grounds of climate warming and increased nutrient inputs due to anthropogenic activities, harmful cyanobacterial blooms (HCBs) become a severe hazard for freshwater ecosystems [1,2,3,4]. Due to the critical economic and public health issues caused by HCBs, extensive research on this topic has been conducted aiming to disclose the detrimental effects of HCBs and mitigation strategies. Recent research has been focused on the strategies applied in HCBs control including chemical, physical, mechanical, and biological methods [5,6,7]. Cyanobacteria blooms are controlled with ultrasound, artificial mixing, and ultraviolet irradiation in the case of physical and mechanical strategies [8,9,10,11]. Chemical products such as photosensitizers (molecules that can be activated by light in order to generate reactive oxygen species which may damage cell structures), metals, and other chemical molecules are among the most commonly used chemical methods [12,13,14]. The introduction of grazers and competitors of cyanobacteria, such as zooplankton, microorganisms (viruses, pathogenic bacteria, or fungi), and macrophytes have been proposed as the most popular biomanipulation for the bio-control of toxic cyanobacteria [15,16,17,18,19]. However, the application of these strategies is not recommended because of their unforeseen ecological consequences, high costs, energy-intensive, and low efficiency [20]. In order to develop effective anti-HCBs agents that are more eco-friendly to the environment, scientists are looking for natural substances released by other aquatic organisms with activity against the growth of cyanobacteria. The marine environment is an excellent source of natural bioactive compounds with unique structures, different from those found in terrestrial natural compounds [21]. Among marine organisms, seaweeds produce active secondary metabolites with a wide range of biological activities, including antibacterial, antifungal, antioxidant, and anti-cyanobacterial compounds [7].

Owing to its specific geographical position, from the Mediterranean Sea to the North and the Atlantic Ocean to the West, Morocco holds a large bio-ecological diversity of seaweeds. This diversity was documented for instance by Chalabi et al. (2015) [22] who reported a particular richness of 489 species distributed at the Mediterranean Coast (381 species) and the Atlantic Coast (323 species). Several compounds with anti-cyanobacterial properties have been purified from the extracts of some seaweed species, such as palmitelaidic acid and 2,3 dihydroxypropyl ester extracted from the methanol extract of *Ulva prolifera* [6], and gossonorol and margaric acid purified from *Gracilaria lemaneiformis* ethanolic extract [23]. Currently and according to our knowledge, unlike the essential oils of plants and plant parts that have been evaluated for their potential anti-cyanobacterial properties, there is no scientific report describing the anti-prokaryotic activity of the essential oils from seaweeds [24,25,26,27,28]. In this respect, the present study aims to uncover for the first time the possible inhibitory effects of essential oils (EOs) extracted from three Moroccan seaweeds that are broadly known by their antimicrobial activities namely *Cystoseira tamariscifolia*, *Sargassum muticum*, and *Ulva lactuca*, on the growth of *Microcystis aeruginosa* a cyanobacteria species that commonly form HABs in Moroccan freshwaters. In addition, this study also provides first insights regarding the anti-cyanobacterial mechanism of EOs, analyzing the growth inhibition power through the following indicators: measurement of chlorophyll-*a*, protein contents, and activity of cellular stress response enzymes of the stated strain.

## 2. Results

### 2.1. Chemical Composition of Moroccan Seaweed EOs

The EO total content based on the dry weight of the seaweed materials are presented in Table 1. The highest total content was achieved with the EO extracted from the green macroalgae *U. lactuca* (0.187% ± 0.078%) followed by the brown seaweed *S. muticum* EO (0.106% ± 0.017%). While, the lowest total content was recorded by the brown seaweed *C. tamariscifolia* EO (0.062% ± 0.018%).

The chemical composition of seaweed EOs was identified qualitatively and quantitatively by GC-MS analysis. The content, expressed in percentage, of the individual components of each seaweed, and retention indices, are summarized in Table 2. Forty constituents were determined in *C. tamariscifolia* EO, corresponding to 59.6% from the total compounds in this species. The major components in this EO were palmitic acid (7.7%) followed by dihydroactinidioide (6.57%), hexahydrofarnesyl acetone (5.1%), heptadecane (4.14%), and phytol (4.1%), while other compounds were present below 4%. In total, 41 compounds (corresponding to 45.7% from the total compounds in this species) were identified in the total *S. muticum* EO composition, the most predominant compounds were found to be palmitoleic acid (7.8%), dihydroactinidiolide (6.97%) and benzeneacetaldehyde (4.62%). Whereas, the EO extracted from *U. lactuca* revealed the presence of the highest number of compounds; 45 compounds (corresponding to 55% from the total compounds in this species), with dominance of eicosapentaenoic acid (8%), dihydroactinidioide (7.8%), and β-ionone (7.6%).

### 2.2. Screening of Anti-Cyanobacterial Activity

**Figure 1:**
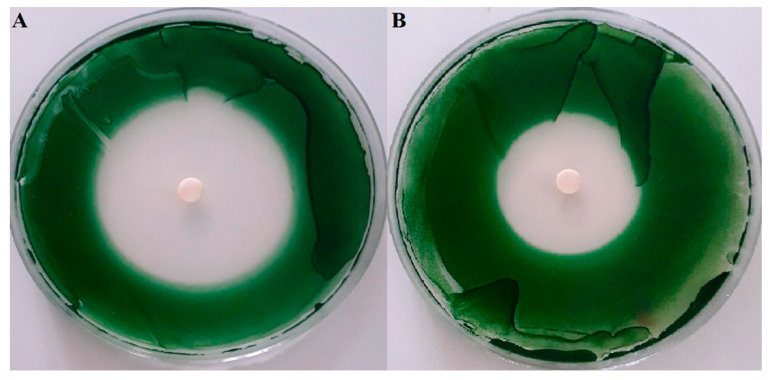
Anti-cyanobacterial activity of the active tested EOs against *M. aeruginosa* on solid media. (**A**) *C. tamariscifolia*; (**B**) *Sargassum muticum*.

The potential anti-cyanobacterial properties of seaweed EOs was evaluated qualitatively using the disk diffusion methods. After 1 week of incubation, the inhibition zones were measured and the results are presented in Table 3 and Figure 1. The results show that the tested green macroalgae *U. lactuca* did not reveal any inhibitory activity against *M. aeruginosa*, while both brown algae showed algicidal activity against the tested cyanobacteria. The most relevant activity was observed with *C. tamariscifolia* EO with zones of inhibition greater than 46 mm. Notably, the growth inhibition of *C. tamariscifolia* EO was approximately similar to the positive control, copper sulphate (CuSO_4_), that presented a growth inhibition diameter of 45.3 mm. Furthermore, *S. muticum* EO showed moderate activity (zone of inhibition was 32 mm). The activity of seaweed EOs against *M. aeruginosa* was determined quantitatively by means of broth microdilution technique. Calculation of minimal inhibitory concentrations (MIC) and minimal bactericidal concentrations (MBC) was performed after 1 week of incubation and the results are summarized in Table 4. CuSO_4_ as positive control displayed a high potency (MIC = MBC = 3.12 μg mL^−1^). The greatest effectiveness was achieved with the EO extracted from *C. tamariscifolia*, with MIC being equal to 7.81 μg mL^−1^ and the MBC equal to 15.62 μg mL^−1^. Whereas, *S. muticum* EO showed a moderate potency with MIC and MBC values of 62.5 and 125 μg mL^−1^, respectively.

### 2.3. Physiological Effects of C. tamariscifolia EO on M. aeruginosa

#### 2.3.1. Inhibitory and Growth Rates of *C. tamariscifolia* EO on Tested Cyanobacteria

**Figure 2:**
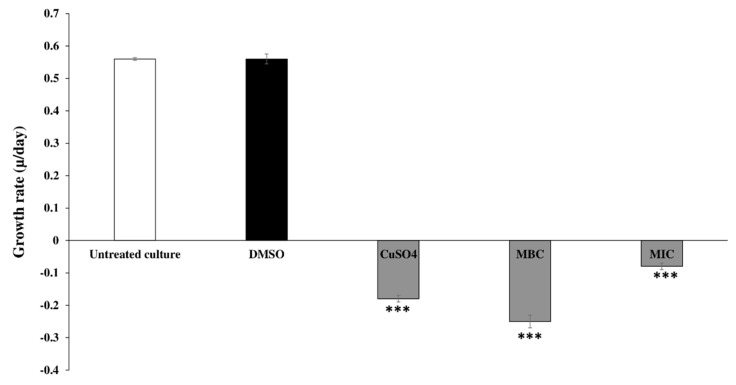
Effect of MIC and MBC, *C. tamariscifolia* EO on the growth rate of *M. aeruginosa*. MIC: minimum inhibitory concentration and MBC: minimum bactericidal concentration. Each value representing mean ± SD of three replicates. *** *p* < 0.001 indicate significant differences compared with the untreated culture.

The physiological effects caused by the action of the essential oil on the cyanobacteria cells were accessed only for the seaweed EO that showed the highest activity on the qualitative assay (Table 3 and Figure 1). The algicidal effects of *C. tamariscifolia* EO at the MIC and MBC concentrations (7.81 and 15.68 μg mL^−1^, respectively) against *M. aeruginosa* are shown as the inhibition and growth rates in Table 4 and Figure 2. The results indicate that *C. tamariscifolia* EO had a significant inhibitory effect on the tested cyanobacteria compared with the negative control (DMSO), which did not show any inhibitory effect. On the first day of the experience, *C. tamariscifolia* EO showed strong growth inhibition for both tested concentrations in a concentration-dependent way. The IR was 67.95% ± 0.38% and 73.93% ± 0.98% at the MIC (7.81 μg mL^−1^) and MBC (15.68 μg mL^−1^), respectively. Thereafter, the inhibition rates increased and set at more than 85% along the experiment, for both used concentrations. The maximum IR was recorded at the last day of treatment with IR of 99.24% ± 0.07% at the MBC concentration. The generation time of *M. aeruginosa* was 1.23/day with the growth rates of 0.75/day under standard culturing conditions and negative control treatment. Under treatment with CuSO_4_, the growth rate and the generation time of the tested cyanobacteria decreased significantly with μ value of −0.18/day and generation time of −3.77/day. The *C. tamariscifolia* EO revealed a strong effect on the growth of *M. aeruginosa* at both tested concentration (the μ value was less than −0.08/day).

#### 2.3.2. Morphological Changes of *M. aeruginosa* Cells

**Figure 3:**
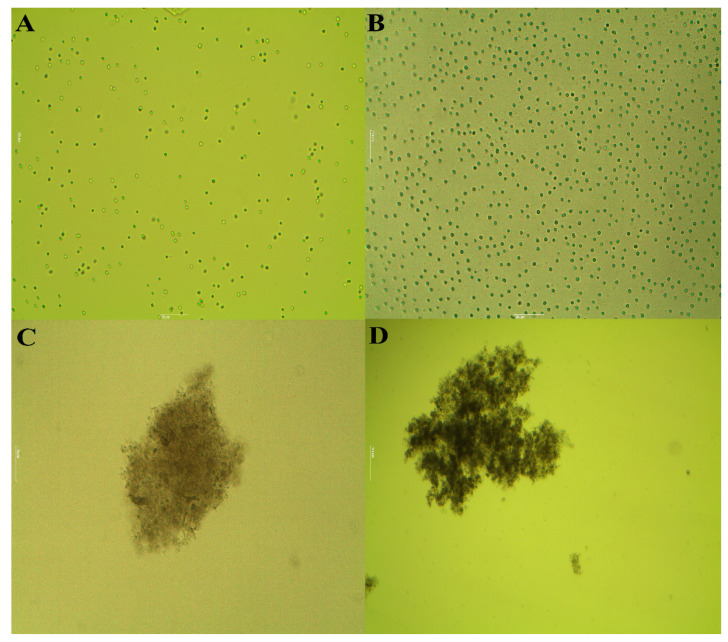
Micrographs of *M. aeruginosa* (Gr. × 40) (**A**) At the first day of treatment; (**B**) untreated culture at the end of treatment; (**C**) culture under treatment with *C. tamariscifolia* EO at second day of the experience; (**D**) culture under treatment with CuSO_4_ at second day of the experience.

The visual observation of the tested unicellular *M. aeruginosa* cultures under *C. tamariscifolia* EO at both used concentrations showed that after 3 days of exposure, a blue color appeared in the treated groups and became colorless after the fifth day of treatment. While, microscope observation at a magnification of ×40 showed that the unicellular cyanobacteria strain becomes colonial on the second day of treatment under stress with *C. tamariscifolia* EO. Moreover, similar morphological changes were also observed under treatment with the positive control (CuSO_4_) (Figure 3).

#### 2.3.3. Effects of *C. tamariscifolia* EO on *M. aeruginosa* Chlorophyll-*a* and Protein Contents

**Figure 4:**
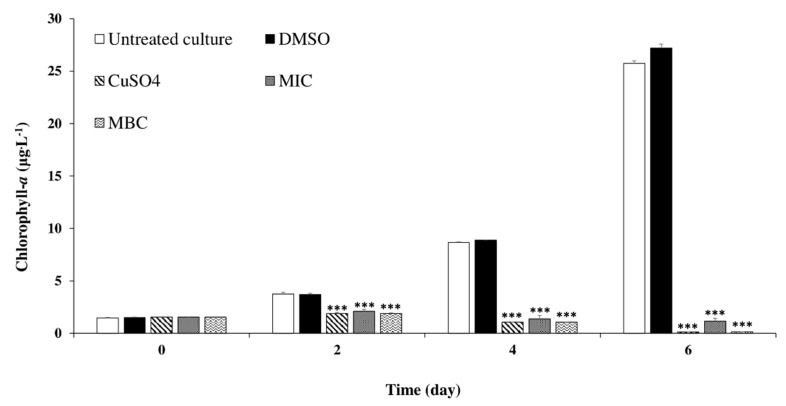
Effect of *C. tamariscifolia* EO on *M. aeruginosa* chlorophyll-*a* concentration. Results are presented as mean ± SD of three independent assays (*** indicates *p* < 0.001 relative to the untreated culture by ANOVA).

To explore whether chlorophyll-*a* synthesis of the treated cells was inhibited by *C. tamariscifolia* EO, the chlorophyll-*a* content of *M. aeruginosa* cells after exposure to *C. tamariscifolia* EO are shown in Figure 4. At the first day of experience, the chlorophyll-*a* content was the same at all treatments (including control). With exposure time, the chlorophyll-*a* content increased significantly for the untreated culture and the negative control. While, *M. aeruginosa* chlorophyll-*a* content was significantly decreased by both used concentrations of *C. tamariscifolia* EO. Compared to CuSO_4_ used as positive control, *C. tamariscifolia* EO recorded a similar effect on *M. aeruginosa* chlorophyll-*a* content during the 6 days of experiment.

**Figure 5:**
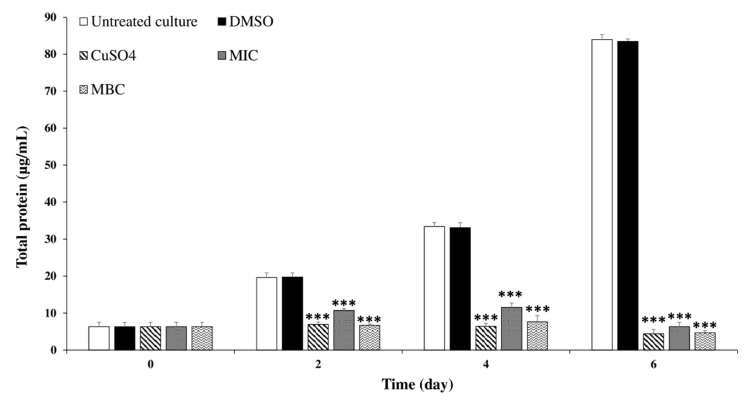
Effect of *C. tamariscifolia* EO on *M. aeruginosa* protein content. Results are presented as mean ± SD of three independent assays (*** indicates *p* < 0.001 relative to the untreated culture by ANOVA).

To investigate the effects of *C. tamariscifolia* EO on *M. aeruginosa* cells, the protein content of *M. aeruginosa* cells was determined after exposure to *C. tamariscifolia* EO, and the results are shown in Figure 5. Similar to chlorophyll-*a*, for the untreated culture and the negative control, the protein content increased significantly with exposure time. On day 2, the differences in protein content for the MIC and MBC concentrations of *C. tamariscifolia* EO were significantly different, compared with the negative control (untreated culture and DMSO). Similar results were recorded on days 4 and 6, *C. tamariscifolia* EO (at both used concentrations) and CuSO_4_ (positive control) recorded a significant reducing effect on the protein content of *M. aeruginosa* cells. Furthermore, the protein contents were highly coherent with the cell density and the chlorophyll-*a* results.

#### 2.3.4. Effects of *C. tamariscifolia* EO Superoxide Dismutase (SOD) and Catalase (CAT) Activities and Malondialdehyde (MDA) Concentration in *M. aeruginosa* Cells

**Figure 6:**
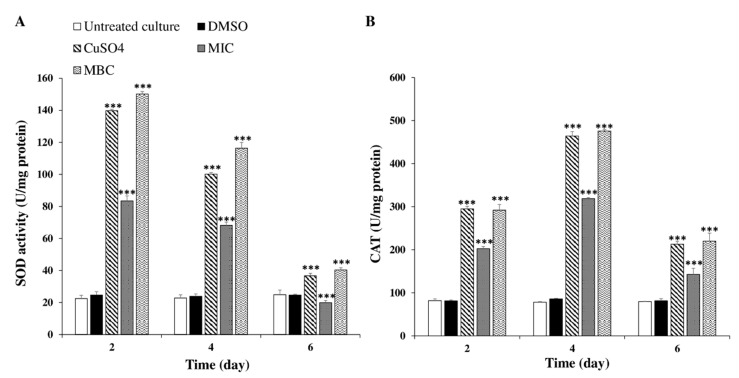
Superoxide dismutase (SOD) (**A**) and catalase (CAT) (**B**) activities in *M. aeruginosa* cells after treatment with *C. tamariscifolia* EO. Results are presented as mean ± SD of three independent assays (*** indicates *p* < 0.001 relative to the untreated culture by ANOVA).

In order to determine whether the cellular oxidative defense system was activated, the superoxide dismutase (SOD) activity, as the first defense against Reactive Oxygen Species (ROS) among antioxidant systems, which catalyzes the superoxide anion into H_2_O_2_ and O_2_, was investigated (Figure 6A). The results demonstrate that the differences in the SOD activities in cyanobacterial cells between control and treatment groups were significant and visible from the second day of treatment. From the second day of experiment, the SOD activity under the MBC concentration (15.62 μg mL^−1^) of *C. tamariscifolia* EO treatment became higher than the positive control (CuSO_4_) and reached the peak of 150.25 U/mg protein. Thereafter, the SOD activity began to decrease gradually in the following time in the control and treatment groups.

The second defense against reactive oxygen species (ROS) is catalase (CAT), which can convert H_2_O_2_ into H_2_O and directly eliminates H_2_O_2_ in the peroxisome. As shown in Figure 6B the CAT activity in *M. aeruginosa* cells exposed to *C. tamariscifolia* EO exhibited a significant increase, while the CAT activity in the untreated culture and the negative control remained unchanged over time. The differences between treated and control groups were apparent from the second day of treatment. The CAT activity at 15.62 μg mL^−1^ of *C. tamariscifolia* EO (MBC concentration) was higher than that at 7.81 μg mL^−1^ of *C. tamariscifolia* EO (MIC concentration); however, the differences between them were not significant. After 4 days of exposure, the activity of CAT greatly increased and reached a maximum value with the exposure to 15.62 μg mL^−1^ *C. tamariscifolia* EO. At the sixth day of treatment, the CAT activity with *C. tamariscifolia* EO decreased but was still significantly different compared with the untreated culture.

**Figure 7:**
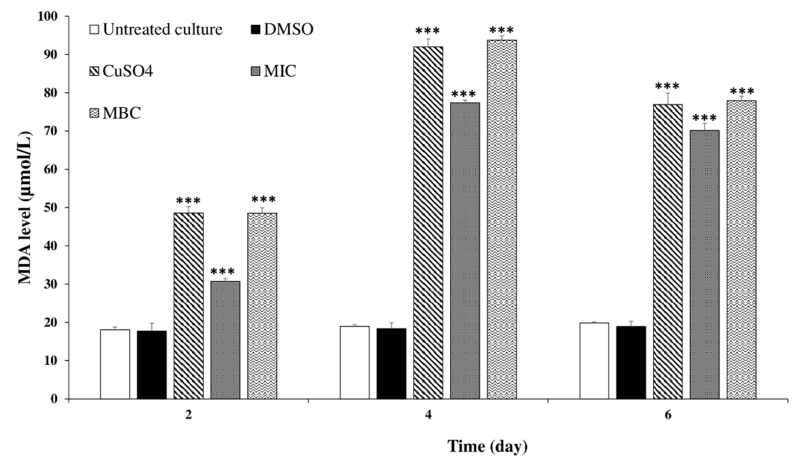
Malondialdehyde (MDA) concentration in *M. aeruginosa* cells after treatment with *C. tamariscifolia* EO. Results are presented as mean ± SD of three independent assays (*** indicates *p* < 0.001 relative to the untreated culture by ANOVA).

Malondialdehyde (MDA, the final product of lipid peroxidation) was used as an indicator of lipid peroxidation, as indicated in Figure 7, the MDA content increased significantly at both tested concentrations of *C. tamariscifolia* EO from the second day of treatment. While, the MDA level in the negative control groups remained unchanged over time. After 48 h of exposure, 15.62 and 7.81 μg mL^−1^ *C. tamariscifolia* EO induced an increase in MDA compared with the control. The MDA concentrations increased with the increased concentration of *C. tamariscifolia* EO. The maximal MDA value was 94 µmol/L at day 4 of treatment with 15.62 μg mL^−1^ *C. tamariscifolia* EO, which was 4.95 times higher than that in the negative control groups.

## 3. Discussion

Seaweeds are one of the most primitive and dominant organisms in aquatic ecosystems. They could provide an eco-friendly approach for HCBs control due to their ability to produce a large range of bioactive compounds [7,29,30]. Therefore, we proceeded to assess the anti-cyanobacterial activity of three Moroccan seaweed EOs. To the best of our knowledge, the present study constitutes the first attempt to extract and characterize Moroccan seaweed EOs. The total content percentage of Moroccan seaweed EOs was lower compared to that reported previously by Patra et al. (2017a, 2015) and Patra and Baek (2016a, 2016b) [31,32,33,34] who found that the total content of seaweeds collected from the Korean coast was usually higher than 0.26%. This difference in seaweed total content percentage could be due to the geographical locations, the species used, harvesting time, and the used extraction method. An important richness and variability of compounds was observed after seaweed EOs chemical analysis. Among the total of 91 compounds identified in the three selected algal EOs, 14 compounds showed to belong to the terpenes group. In general, terpenoids are compounds that have been associated with several bioactive properties, including antimicrobial activity [35].

The most abundant constituents in the two brown seaweeds *C. tamariscifolia* and *S. muticum* EOs were fatty acids, namely palmitic acid and palmitoleic acid, respectively. Previous studies on EOs from other species of seaweeds have shown the presence of hexadecenoic acid (palmitoleic acid). In particular, Patra et al. (2017a) [36] reported the presence of this unsaturated fatty acid as one of the main compounds (22.39%) of the brown edible seaweed *Undaria pinnatifida* EO collected from the Korean coast. A high content of palmitic acid (9.2% and 16.57%) was also found by Patra et al. (2017b, 2015a) [31,37] on *Porphyra tenera* and *Laminaria japonica* EOs, respectively. Previously, the EO composition of seaweed species of *Cystoseira* genus, other than *C. tamariscifolia*, has been described. Ozdemir et al. (2006) [38] reported that the brown seaweed *C. barbata* EO (*Cystoseira* genus) consists of several compounds different from those we have determined in *C. tamariscifolia* EO, such as docosane (7.61%), tetratriacontane (7.47%), eicosane (5.05%), tricosane (4.43%), hexadecane (4.16%), and heptadecane (1.35%) as major compounds. Additionally, 1-chloro-2,2-diethoxyethane (21.5%), 2,3-butanediol (6.5%), chloroacetic acid (3.7%), and 1,1-dichloro-2,2-diethoxyethane (2%) were identified in the volatile compounds composition of *C. crinite* (*Cystoseira* genus) collected from the eastern Mediterranean [39]. Nevertheless, it should be noticed that research on the chemical composition of seaweed EOs is still very scarce. Regarding the chemical composition of the green macroalgae *U. lactuca* EO, the polyunsaturated fatty acid eicosapentaenoic acid (8%) was the most dominant constituent. The presence of other polyunsaturated fatty acids, such as cis- and trans-5,8,11,14-eicosatetraenoic acids were also detected in the *Dictyopteris polypodioides* EO collected from the Algerian coast [40].

Among the identified compounds, some terpenes such as β-caryophyllene (0.02%) and α-pinene (0.015%) were only found in minor amounts, while others such as dihydroactinidiolide (6.6–7.8%), β-Ionone (not detected to 7.6%), and phytol (0.23–4.1%) were present in higher amounts. Terpenes such as safranal and others have been recently reported in the essential oil extracted from the brown algae *D. polypodioides* [40]. Similarly, the presence of the terpenic compounds dihydroactinidiolide, β-Ionone and phytol has been previously described in marine algae [41]. Besides terpenes, several compounds belonging to different groups such as alcohols, aldehydes, and ketones were present in minor amounts. The presence of such compounds is in good agreement with Gressler et al. (2009) [41], who mentioned the ability of marine algae to produce a wide range of metabolites including hydrocarbons, terpenes, fatty acids, esters, alcohols, aldehydes, and ketones.

The qualitative screening using the paper disk diffusion method in solid medium demonstrated that the green macroalgae *U. lactuca* did not show any inhibitory activity against the tested Gram-negative bacteria *M. aeruginosa*. These results are in good agreement with those found by Zerrifi et al. (2019) [30] who investigated the anti-cyanobacterial activity of *U. lactuca* methanolic extract collected from the Moroccan coast. Their results showed that the *U. lactuca* extract also did not have an effect against *M. aeruginosa*. Similar results were obtained by Salvador et al. (2007) [42] who reported that the seaweeds of the genus *Ulva* did not show antibacterial activity against any assayed Gram-negative bacteria. On the contrary, Mishra (2018) [43] observed that methanol, butanol, and ethyl acetate extract of *U. lactuca* display moderate activity against *Pseudomonas aeruginosa*. Additionally, Begum et al. (2018) [44] reported that the methanolic extract of *U. reticulate* (*Ulva* genus) revealed the maximum inhibitory activity against Gram-negative bacteria *P. aeruginosa*. Furthermore, we found that *S. muticum* EO showed a strong activity against *M. aeruginosa* (32.33 mm). Our results are in agreement with those found by Kumaresan et al. (2018) [45] who investigated the antimicrobial activity of *S. wightii* (*Sargassum* genus) aqueous extract and showed that this extract had an important antibacterial activity against Gram-negative bacteria *Escherichia coli* with inhibition zone of 13 mm. Sujatha et al. (2019) [46] observed that *S. swartzii* ethanolic extract exhibited a high antibacterial activity against Gram-negative bacteria. *S. muticum* extract was active against *Enterobacter aerogenus, Proteus vulgaris, Salmonella typhymurium,* and *Salmonella paratyphi* [47]. In what concerns the activity against *M. aeruginosa*, Zerrifi et al. (2019) [30] evaluated the methanolic extract of *S. muticum*, which did not reveal any inhibiting capacity, most probably due to the different chemical composition between the methanolic extract and that obtained by hydrodistillation. As mentioned, in the present study the highest activity was recorded for *C. tamariscifolia* EO (46.33 mm). However, this finding is in disagreement with those found by Farid et al. (2009) [48]. These authors investigated the antibacterial activities of *C. tamariscifolia* collected from Morocco, and their results suggest that *C. tamariscifolia* dichloromethane/methanol extract did not show antimicrobial activity against the Gram-negative bacteria assayed (*E. coli*). Salvador et al. (2007) [42] observed that *C. tamariscifolia* extract did not inhibit the growth of any tested Gram-negative bacteria. On the other hand, Ozdemir et al. (2006) [38] reported that the volatile oil of the genus *Cystoseira* recorded a moderate effect on the tested Gram-negative bacteria (7 mm against *E. coli* and *S. typhimurium*). Contrarily, Zerrifi et al. (2019) [30] observed that *C. tamariscifolia* extract conferred an important activity against Gram-negative bacteria *M. aeruginosa* with inhibition zone equal to 13.33 mm. In another report, Chiheb et al. (2009) [49] reported that different *Cystoseira* species show a potent antibacterial activity against tested Gram-negative bacteria *Salmonella typhi, E. coli, P. aeruginosa*, and *Klebsiella* sp. Ainane et al. (2014) [50] found that *C. tamariscifolia* extract produce interesting zones of inhibition against both tested Gram-negative bacteria, *Enterobacter cloacae* and *Klebsiella pneumoniae* (inhibition diameter between 10 and 15 mm). Likewise, a recent study showed the high antibacterial activity against Gram-negative bacteria of another species of the *Cystoseira* genus (*C. mediterranea*) [51].

The quantitative screening using the broth microdilution technique confirmed the results of the qualitative assay since *C. tamariscifolia* EO achieved the greatest effectiveness against *M. aeruginosa* (MIC = 7.81 μg mL^−1^ and MBC = 15.62 μg mL^−1^). These results are in accordance with those reported earlier by Wang et al. (2015, 2014) and Zerrifi et al. (2020) [27,28,52] who found that other EOs also showed a high activity against the toxic cyanobacteria *M. aeruginosa*. Our results in the liquid medium revealed that *C. tamariscifolia* EO recorded a significant anti-cyanobacterial activity against the toxic cyanobacteria *M. aeruginosa* with a percentage inhibition of more than 67%. Several studies were conducted to investigate growth inhibition by EOs extracted from many aquatic and terrestrial plants and solvent extract of seaweeds on *M. aeruginosa*. Wang et al. (2014) [52] reviewed the anti-cyanobacterial activity of two emergent plant EOs (*Typha latifolia* and *Arundo donax*) on *M. aeruginosa*. The authors reported inhibition rates of more than 40% at 50.0 mg L^−1^ of both tested EOs. The *Rosmarinus officinalis* EO recorded significant growth inhibition against *M. aeruginosa* [25]. Moreover, Wang et al. (2015) [27] showed that the growth of *M. aeruginosa* was strongly inhibited by *Vallisneria spinulosa* EO at 50.0 mg L^−1^ with an inhibition rate equal to 41.7%. Xian et al. (2006) [53] found that *Ceratophyllum demersum* EO composed of fatty compounds, terpenoids, and phenolic compounds, recorded a high inhibitory activity on *M. aeruginosa* growth. Furthermore, Zerrifi et al. (2019) [30] tested the effect of *C. tamariscifolia* methanolic extract on the growth of *M. aeruginosa*. Their results show that the reached inhibition rates were higher than 49% after the first day of treatment at 0.6 mg L^−1^. The morphological changes observed in *M. aeruginosa* culture in this study are quite similar to those observed by Harada et al. (2009), Huang et al. (2002), and Zerrifi et al. (2020) [28,54,55]. The chlorophyll-*a* and protein content that reflect *M. aeruginosa* growth, was decreased after *C. tamariscifolia* EO treatment. This decrease could be related to malfunctions of normal physiological metabolism in cyanobacterial cells (e.g., disruption of Photosystem I and destruction of Photosystem II) [56,57]. The mentioned changes can be related to the chemical composition of *C. tamariscifolia* EO, namely to the presence, although in low amounts, of several terpenoids, including oxygenated compounds such as alcohols and aldehydes. Besides, the possibility of synergisms, between the more abundant terpenoids, such as dihydroactinidiolide (6.57%) and hexahydrofarnesyl acetone (5.1%) with compounds present in minor amounts, should also be considered. Finally, all the three studied seaweeds presented a very complex composition, with a large abundance of compounds, several of which were not possible to be identified by the used technique (GC-MS). Some of those unidentified compounds can possibly explain the different activity observed for the three samples.

The activation of SOD and CAT activities were responsible for the protection of *M. aeruginosa* cells against oxidative exposure (eliminate ROS or reduce damaging effects). Our finding of SOD and CAT activities in *M. aeruginosa* were also observed in response to glyphosate treatment [58]. Meng et al. (2015) [59] observed a significant increase in the SOD activity of *M. aeruginosa* exposed to different concentrations of *Ailanthus altissima* extract. Similar results are also found from other treatments, such as rice straw aqueous extract [60], heptanoic acid and benzoic acid [61], fenoxaprop-*p*-ethyl [62], 17b-estradiol [63], and juglone (5-hydroxy-1,4-naphthoquinone) [64] on *M. aeruginosa*. The last product of lipid peroxidation is MDA, which is an indicator of oxidative stress [65]. The increase of MDA concentration is in agreement with Zhang et al. (2017) [66] who detected a significant increase in MDA levels of *M. aeruginosa* exposed to 5 and 10 mg L^−1^ of glufosinate, comparing to control, 0.5, and 1 mg L^−1^, indicates the occurrence of damage to the lipid membranes. The treatment of *M. aeruginosa* cells with pyrogallol (polyphenol) caused lipid peroxidation and altered MDA levels [67]. Contrariwise, Xie et al. (2019) [68] found that the MDA concentrations on *M. aeruginosa* cells showed no apparent change under napropamide and acetochlor treatments.

## 4. Conclusions

After screening of EOs extracted from Moroccan seaweeds for their anti-cyanobacterial activity, our results revealed that marine macroalgae EOs are potential producers of anti-cyanobacteria compounds. Consequently, they should be subject to a comprehensive study as natural sources of bioactive substances. Accordingly, to better understand the potential effects and the mechanisms of action of the studied EOs on *M. aeruginosa*, the search of the active EOs major components effects on *M. aeruginosa* will be the next step of our research. Moreover, further research will need to be conducted using other seaweeds and/or phytoplankton species in macrocosms and natural field conditions, studying the toxicity, nature, and stability of the compounds and their potentially synergistic interactions in the aquatic ecosystem.

## 5. Materials and Methods

### 5.1. Seaweed Material Sampling and Extraction of Essential Oils (EOs)

Three seaweed species were selected for the study: *C. tamariscifolia* (Phaephyceae, Sargassaceae), *S. muticum* (Phaephyceae, Sargassaceae), and *U. lactuca* (Ulvophyceae, Ulvaceae). These macro-algae were harvested from two Moroccan coastal regions (Table 5).

The samples were rinsed with seawater and distilled water to remove debris. After identification of each species according to their morphological and histological features [69], seaweed materials were dried in the shade at room temperature (≈25 °C) and subjected to hydro-distillation, using a Clevenger-type apparatus for 3 h until total recovery of oil. The EOs obtained were dried over anhydrous sodium sulfate and stored at 4 °C in the dark.

### 5.2. Gas Chromatography/Mass Spectrometry (GC/MS) Analyses

The seaweed essential oils were analyzed by GC-MS following a protocol previously described by Falcão et al. (2018) [70]. Analyses were performed in a GC-2010 Plus (Shimadzu, Kioto, Japan) gas chromatography system equipped with a AOC-20iPlus (Shimadzu, Kioto, Japan) automatic injector, a SH-RXi-5ms column (30 m × 0.25 mm × 0.25 μm; Shimadzu, Kioto, Japan), and a mass spectrometry detector, operated using an injector temperature of 260 °C and the following oven temperature profiles: an isothermal hold at 40 °C for 4 min, an increase of 3 °C/min to 175 °C, followed by an increase of 15 °C/min to 300 °C and an isothermal hold for 10 min. The transfer line temperature was set at 280 °C and the ion source at 220 °C; the carrier gas, helium, was adjusted to a linear velocity of 30 cm/s; the ionization energy was 70 eV, the scan range was set at 35–500 u, with a scan time of 0.3 s. A quantity of 1 μL of each sample diluted in n-hexanewas injected using the split injection mode at 1:10. The identification of the essential oil components was carried out by comparison of the obtained spectra with those from the NIST17 mass spectral library and by determining the linear retention index (LRI) based on the retention times of an n-alkanes mixture (C8–C40, Supelco, Darmstadt, Germany). When possible, comparisons were also performed with commercial standard compounds and with published data. Compounds were quantified as relative percentage of total volatiles using relative peak area values obtained from total ion current (TIC).

### 5.3. Screening for Anti-Cyanobacterial Activity

#### 5.3.1. Cyanobacteria Strain

In this study, the cyanobacteria strain *M. aeruginosa* was sampled from the eutrophic reservoir Lalla Takerkoust (31°21′36″ N; 8°7′48″ W), Morocco, in bloom period (October 2017) and then the strain was isolated, separated into single cells, and maintained in culture in BG11 medium under a controlled culture chamber endowed with the following conditions: temperature of 26 ± 2 °C, light intensity of 63 μmol m^−2^ s^−1^, and a light/dark cycle of 15 h/9 h [30].

#### 5.3.2. Disc Diffusion Method

In vitro anti-cyanobacterial activity of seaweed EOs of each of the three algae was evaluated using the agar diffusion method [71]. The suspension of tested *M. aeruginosa*, containing about 10^8^ cells/mL using a Malassez counting cell, was spread on BG11 medium with 4% of agarose. Subsequently, 10 μL of each EO and CuSO_4_, prepared at a concentration of 50 μg mL^−1^ in ultrapure water, as positive control was dropped on sterile filter paper discs, 6 mm in diameter (Whatman no. 1, Little Chalfont, UK) and placed on the agar surface. Before incubation in the culture chamber under the described condition, all treated plates were stored in a refrigerator at 4 °C for more than 4 h to prevent the cyanobacteria growth and allow the diffusion of the bioactive substances contained in the EOs into the medium. Each experiment was repeated six times to statistically confirm the results.

#### 5.3.3. Determination of the Minimum Inhibitory Concentration (MIC) and Minimum Bactericidal Concentration (MBC)

The determination of the MIC values of the EOs that showed activity in the disc diffusion assay, was carried out in a 96-well microplate using the microdilution assay according to the NCCLS guidelines M7-A4 [72]. The MIC values represent the lowest EO concentration that prevents the cyanobacteria growth. Succinctly, 200 μL of tested cyanobacteria culture with density of 3 × 10^6^ cells/mL (exponential growth phase) was added to each microplate well. The EOs were dissolved in DMSO (1%) and added to the tested culture to obtain final concentrations from 4000 to 1.953 μg mL^−1^. Subsequently, the prepared microplates were incubated for 5 days under the described controlled conditions in the culture chamber. In order to determine the MBC values, which represent the lowest EOs concentration that induces 100% cell death of incubated cyanobacteria, 100 μL of each wells without visible cyanobacteria growth was spread on BG11 and incubated for 5 days in the culture chamber.

### 5.4. Determination of Cyanobacteria Growth Rates

The effects of the most bioactive EO on *M. aeruginosa* strain, namely the EO of *C. tamariscifolia*, were accessed by measuring the inhibition and growth rates estimation. The growth test was conducted, in triplicate, under the determined MIC and MBC concentration of the most bioactive EO and CuSO_4_ (positive control) in Erlenmeyer flasks (150 mL) containing 9 mL of cyanobacteria inoculum and 71 mL of BG11 medium. The initial density of the tested cyanobacteria culture was adjusted by addition of BG11 medium and counting cells until a value of 2 × 10^6^ cells/mL (the exponential growth phase). DMSO was employed as negative control. Whereas, another untreated cyanobacteria culture was used for the performance of all calculations necessary for the results treatment. The inhibition (IR) and growth rates were estimated by cells counting using a hemocytometer under a microscope every 24 h [73] and calculated using the following Equations (1) and (2), respectively:

IR(%) = (((Nc − Nt))/(Nc) × 100)(1)

where, Nc and Nt represent the cell concentrations (cells/mL) in the control and treatment samples, respectively [74].

μ = (ln Ne − ln Nb)/Δt(2)

In which µ is the average growth rate; Ne and Nb (cells/mL) are the cell densities on the last day and the first day of the experiment, respectively, and Δt denotes the duration of the experiment.

### 5.5. Biochemical Parameters in M. aeruginosa

#### 5.5.1. Determination of Chlorophyll-*a* and Total Protein Contents

Chlorophyll-*a* (Chl-*a*) concentration was measured in triplicate and calculated following the method previously described by Lichtenthaler and Wellburn (1983) [75]. Shortly, 5 mL of the culture sample was centrifuged at 4000× *g* for 15 min to collect algal cells. Cells were then re-suspended with boiling ethanol (95%). The three replicas were incubated at 4 °C for 48 h. Subsequently, another centrifugation for 5 min at 3400× *g* was performed to eliminate the pellet. The supernatant optical density (OD) was read at different wavelengths absorbance (649 and 665 nm). Chlorophyll-*a* concentration was calculated by the following Equation (3).

[Chl-*a*] = 13.95 × DO665 − 6.88 × DO649 (3)

The enzyme extracts were prepared according to Li et al. (2016) protocol [76]. Briefly, *M. aeruginosa* cells were collected by centrifugation of each culture (5 mL) at 4000× *g* for 25 min. The pellet was re-suspended in 0.1 M phosphate buffer (pH 6.5) containing 1% (*w*/*v*) polyvinylpyrrolidone (PVP). Then the cells were disrupted and homogenized by an ultrasonic cell pulverizer for 5 min in an ice bath. The homogenate was then centrifuged 10,000× *g* at 4 °C for 10 min. The supernatant was used for total protein measurement and antioxidant enzyme activity assays. The total protein content was determined by the application of Bradford (1976) method [77]. Briefly, 100 μL of the enzyme extract was added to 2 mL of Bradford’s reagent and incubated at room temperature in obscurity for 20 min. Furthermore, a mixture of the assay buffer (100 μL) and the Bradford’s reagent (2 mL) was used as a blank. The absorbance was read at 595 nm and the protein content was calculated from a calibration curve of Bovine Serum Albumin (BSA).

#### 5.5.2. Activity of Antioxidant Response Enzymes, CAT and SOD

The SOD activity was assayed in triplicate according to Beauchamp and Fridovich (1971) method [78]. The reaction mixture contained 0.8 mL PBS solution (50 mM, pH 7.8), 0.3 mL methionine solution (130 mM), 0.3 mL Na_2_EDTA solution (100 μM), 0.3 mL riboflavin solution (20 μM), 0.3 mL nitroblue tetrazolium (NBT) solution (750 μM), and 1 mL enzyme extract for a total volume of 3 mL. As SOD has the ability to inhibit the photochemical reduction of NBT, this assay utilized negative controls (silver paper wrapped around the test tube to mimic fully dark condition without any photochemical reduction of NBT), positive controls (deficiency of SOD activity in light with full photochemical reduction of NBT), and treatment groups (in light with SOD inhibition on photochemical reduction of NBT). The absorbencies of all experimental tubes were measured at 560 nm after a 20 min irradiance of 40–60 mmol photons m^−2^ s^−1^. One unit of SOD activity was defined as the amount of enzyme that inhibited 50% of photochemical reduction of NBT. CAT activity was assayed in triplicate by absorbance decrease being proportional to the breakdown rate of hydrogen peroxide (H_2_O_2_) at 240 nm according to the method of Rao et al. (1996) [79]. The reaction mixture contained 1 mL H_2_O_2_, 1.9 mL H_2_O, and 1 mL crude enzyme. Samples were incubated for 2 min at 37 °C and the absorbance of the sample was monitored for 5 min at 240 nm using a Varian Cary^®^ 50 UV-Vis Spectrophotometer (Agilent Technologies, Santa Clara, CA, USA).

#### 5.5.3. Determination of MDA Content

The lipid peroxidation level was reflected by changes of malondialdehyde (MDA) content, which was determined in triplicate, according to Du et al. (2017) [62]. Samples were collected every 2 days and centrifuged at 4000× *g* for 20 min. The cell pellets were homogenized with 2 mL of 10% (*w*/*v*) trichloroacetic acid (TCA) and centrifuged at 12,000× *g* for 10 min at 4 °C. After centrifugation, 2 mL of the supernatant was mixed with 2 mL of 0.6% thiobarbituric acid (in 10% TCA) and heated in boiling water for 15 min. The reaction was stopped by transferring the reaction tubes into an ice bath. Following cooling, the samples were then centrifuged at 12,000× *g* for 10 min. The absorbance of the supernatant was measured at 532, 600, and 450 nm, taking a mixture of 2 mL ultrapure water and 2 mL 0.6% TBA as reference. The MDA level (μmol/L) was calculated according to Equation (4):

MDA = 6.45 × OD532 − OD600 − 0.56 × OD450(4)

### 5.6. Statistical Analysis

The experiments were done in six replicates in solid medium (*n* = 6) and three replicates in liquid medium (*n* = 3) with each independent assay. Statistical analysis between experimental groups and the control were performed by applying a one-way and two-way ANOVA analysis. Post hoc differences between group means was carried out with the Tukey test using Sigma Plot software (sigmaplot 12.5 for windows; Systat Software Inc., San Jose, CA, USA) for Windows. Values of *p* < 0.001 were considered statistically significant.

## Figures and Tables

**Table 1:** Total content of studied seaweed essential oils.

Species	EO Total Content (%, *v*/*w*)
*Ulva lactuca*	0.19 ± 0.08
*Sargassum muticum*	0.11 ± 0.02
*Cystoseira tamariscifolia*	0.06 ± 0.02

**Table 2:** Chemical composition of essential oils extracted from Moroccan seaweeds (%). Values in bold represent the major compounds present in each sample.

						Relative % ^c^	
Nº	Compound	RT (min)	LRI ^a^	LRI ^b^	Ct	Sm	Ul
1	(E)-2-Pentenal	6.27	750	744	-	-	0.086 ± 0.002
2	4-Methyl-2-pentanol	6.42	754	745	-	-	0.0063 ± 0.0002
3	Toluene	6.69	763	756	-	-	0.143 ± 0.004
4	Hexanal or *n*-Caproylaldehyde	7.92	800	801	0.196 ± 0.001	0.44 ± 0.02	0.23 ± 0.01
5	Furfural	9.24	828	828	0.19 ± 0.01	0.069 ± 0.002	-
6	4-Hexen-3-one	9.41	832	-	-	-	0.075 ± 0.001
7	3-Hexen-2-one	9.73	839	834 *	-	-	0.046 ± 0.003
8	(E)-2-Hexenal	10.17	848	846	0.085 ± 0.003	0.35 ± 0.01	0.347 ± 0.003
9	2-Furanmethanol	10.37	852	853 *	0.57± 0.01	-	-
10	1-Hexanol	11.11	868	863	-	0.063 ± 0.005	0.023 ± 0.001
11	4-Cyclopentene-1,3-dione	11.67	880	880 *	0.6 ± 0.01	0.02 ± 0	-
12	2-Heptanone	12.05	888	889	-	0.029 ± 0.001	0.35 ± 0.01
13	*cis*-4-Heptenal	12.47	897	893	-	0.115 ± 0.005	0.28 ± 0.01
14	*n*-Heptanal	12.57	900	901	0.29 ± 0.01	0.136 ± 0.003	0.293 ± 0.002
15	Acetylfuran	12.99	908	909	0.2 ± 0.005	0.176 ± 0.002	-
16	2-Cyclohexen-1-one	13.98	927	927 *	0.102 ± 0.002	-	-
17	α-Pinene	14.20	931	932	-	0.0151 ± 0.0001	-
18	Cyclohexen-2-one	14.22	931	-	-	-	0.07 ± 0.001
19	Hept-3-en-2-one	14.37	934	927	-	0.038 ± 0.004	0.052 ± 0.003
20	Benzaldehyde	15.00	956	952	0.31 ± 0.01	0.38 ± 0.01	0.569 ± 0.004
21	5-Methyl-furfural	15.71	960	957	0.737 ± 0.001	0.988 ± 0.02	3.39 ± 0.05
22	3,5,5-Trimethyl-2-hexene	16.47	975	-	-	-	0.192 ± 0.004
23	1-Octen-3-ol	16.63	978	974	0.103 ± 0.004	0.2036 ± 0.0001	-
24	2-methyl-3-Octanone	16.91	983	985 *	-	0.318 ± 0.004	-
25	6-Methyl-5-heptene-2-one	16.99	985	986 *	-	0.056 ± 0.004	-
26	3-Methyl-3-cyclohexen-1-one	17.00	985	-	-	-	0.69 ± 0.02
27	Octanal	17.81	1000	998	0.206 ± 0.003	-	-
28	Pyrrole-2-carboxaldehyde	18.12	1006	1008 *	-	0.17 ± 0.01	-
29	(E,E)-2,4-Heptadienal	18.16	1007	1005	0.29 ± 0.01	0.09 ± 0.01	0.466 ± 0.003
30	4-Oxohex-2-enal	19.49	1033	-	1.43 ± 0.03	-
31	2,2,6-Trimethyl-Cyclohexanone	19.56	1034	1036 *	-	-	0.83 ± 0.04
32	Benzeneacetaldehyde	19.93	1041	1036	1.9 ± 0.03	**4.62 ± 0.04**	0.822 ± 0.003
33	γ-Hexalactone	20.39	1050	1047	-	0.42 ± 0.01	1.14 ± 0.01
34	2,4,4-Trimethyl-2-cyclohexen-1-ol	20.42	1051	-	0.65 ± 0.01	-	-
35	(E)-2-Octenal	20.66	1055	1049	0.104 ± 0.001	0.149 ± 0.01	-
36	(R)-3,5,5-Trimethylcyclohex-3-en-1-ol	21.09	1063	-	-	-	0.187 ± 0.003
37	3-Methyl-benzaldehyde	21.27	1067	1064	-	-	0.26 ± 0.01
38	1-Octanol	21.39	1069	1063	0.23 ± 0.004	-	-
39	3,5-Octadien-2-one	22.60	1093	1093	-	-	0.63 ± 0.02
40	Phenylethyl Alcohol	23.49	1110	1115 *	-	1.14 ± 0.02	-
41	Isophorone	23.95	1120	1118	-	-	0.51 ± 0.01
42	4-Oxoisophorone	25.01	1141	1142 *	0.29 ± 0.01	0.813 ± 0.005	0.341 ± 0.01
43	Isomenthone	25.56	1152	1162 *	-	0.485 ± 0.003	-
44	2,6-Nonadienal, (E,Z)	25.61	1153	1150	-	-	0.35 ± 0.005
45	(E)-2-Nonenal	25.81	1157	1157	0.279 ± 0.005	-	-
46	1-Phenyl-1-propanone	26.19	1165	-	-	-	0.49 ± 0.01
47	2,2,6-Trimethyl-1,4-cyclohexanedione	26.33	1167	-	-	-	0.274 ± 0.003
48	2,4-Dimethyl-benzaldehyde	26.67	1175	1175 *	-	-	0.305 ± 0.01
49	1-(4-Methylphenyl)-ethanone	27.02	1181	1182	0.43 ± 0.01	-	-
50	*p*-Methylacetophenone	27.15	1184	1179	-	-	0.54 ± 0.03
51	Safranal	27.82	1198	1197	0.97 ± 0.01	-	1.8 ± 0.1
52	β-Cyclocitral	28.84	1219	1219	-	0.479 ± 0.001	0.614 ± 0.002
53	Ethylmethylmaleimide	29.67	1237	1234	1.562 ± 0.03	3.69 ± 0.03	0.59 ± 0.01
54	Pulegone	29.73	1238	1233	-	0.375 ± 0.003	-
55	2,6,6-Trimethyl-1-Cyclohexene-1-acetaldehyde	30.70	1259	1253 *	-	-	0.53 ± 0.01
56	2,3,6-Trimethyl-7-octen-3-ol	31.53	1277	-	-	1.846 ± 0.01	-
57	Indole	32.47	1297	1290	-	-	1.026 ± 0.003
58	Carvacrol	32.69	1302	1298	0.98 ± 0.02	-	-
59	γ-Amylbutyrolactone	35.29	1361	1362 *	-	1.03 ± 0.02	-
60	Capric acid	35.97	1376	-	1.15 ± 0.02	-	-
61	Fumaric acid, ethyl 2-methylallyl ester	36.27	1383	-	-	1.45 ± 0.03	-
62	β-Caryophyllene	37.91	1421	1417	-	0.02 ± 0.004	-
63	α-Ionone	38.22	1428	1428	3.23 ± 0.02	3.07 ± 0.03	1.1 ± 0.01
64	Nerylacetone	39.27	1454	1434	-	-	0.26 ± 0.01
65	β-Ionone	40.88	1492	1488	1.3 ± 0.03	-	**7.6 ± 0.2**
66	Dihydroactinidiolide	42.49	1536	1538 *	**6.577 ± 0.004**	**6.971 ± 0.003**	**7.8 ± 0.2**
67	Lauric acid	43.66	1562	1565	2.9 ± 0.1	0.49 ± 0.01	1.2 ± 0.1
68	Fumaric acid, ethyl 2-Methylallyl ester	44.46	1583	-	-	-	3.0 ± 0.1
69	Tridecanoic acid	47.61	1666	1662	-	-	0.1979 ± 0.0003
70	3-Keto-β-ionone	47.77	1670	1661 *	-	1.29 ± 0.02	-
71	4-(4-hydroxy-2,2,6-trimethyl-7-oxabicyclo[4.1.0]hept-1-yl)-3-Buten-2-one	48.40	1687	1690	-	1.2 ± 0.1	-
72	Heptadecane	48.77	1697	1700	**4.14 ± 0.04**	-	-
73	Pentadecanal	49.37	1711	1713	-	-	0.27 ± 0.01
74	Myristic acid	50.61	1769	1765 *	2.2 ± 0.1	2.16 ± 0.01	1.855 ± 0.001
75	Pentadecanoic acid	51.81	1820	1869	-	-	0.121 ± 0.004
76	Hexahydrofarnesyl acetone	52.07	1847	-	**5.1 ± 0.1**	-	-
77	2-Pentadecanone, 6,10,14-trimethyl	52.11	1843	1847	-	-	0.23 ± 0.01
78	Methyl 4,7,10,13-hexadecatetraenoate	52.68	1885	-	-	-	0.15 ± 0.01
79	Eicosane	52.80	1895	-	0.22 ± 0.02	-	-
80	Palmitoleic acid	53.41	1948	1953*	-	**7.8 ± 0.1**	-
81	Eicosapentaenoic acid	53.57	1962	-	-	-	**8.0 ± 0.2**
82	Palmitic acid	53.64	1968	1959	**7.7 ± 0.1**	0.73 ± 0.01	2.887 ± 0.02
83	Phytol	55.00	2113	2111 *	**4.1 ± 0.1**	0.38 ± 0.03	0.23 ± 0.01
84	Linolenic acid	55.36	2159	2134 *	-	-	1.2 ± 0.1
85	Eicosanal	55.83	2223	2224	0.7 ± 0.1	-	-
86	1-Hexacosanol	56.25	2283	2906	1.39 ± 0.05	-	-
87	Henicosanal	56.52	2325	2329	0.89 ± 0.04	-	-
88	Docosanal	57.15	2427	2434	1.38 ± 0.04	-	-
89	1-Docosanol	57.51	2488	2470	2.423 ± 0.004	-	-
90	Tricosanal	57.75	2529	2534	2.8 ± 0.1	-	-
91	Bis (2-ethylhexyl) phthalate	57.95	2562	2550 *	-	-	0.5 ± 0.1
	Total identified (%)				59.6 ± 0.1	45.7 ± 0.1	55 ± 1
	Not identified (%)				40.4 ± 0.1	54.3 ± 0.1	45 ± 1

^a^ LRI, linear retention index determined on a DB-5 MS fused silica column relative to a series of *n*-alkanes (C8–C40). ^b^ Linear retention index reported in literature (Adams, 2017). ^c^ Relative % is given as mean ± SD, *n* = 3. * NIST Standard Reference Database 69: NIST Chemistry WebBook. Ct. *Cystoseira tamariscifolia*; Sm. *Sargassum muticum*; Ul. *Ulva lactuca*.

**Table 3:** Inhibition-zone diameters, minimal inhibitory concentrations (MIC) and minimal bactericidal concentrations (MBC) of Moroccan seaweed essential oils (EOs).

Treatments	Inhibition Zone (mm)	MIC (μg mL^−1^)	MBC (μg mL^−1^)
*C. tamariscifolia*	46.3 ± 0.6 ***	7.81	15.62
*S. muticum*	32.3 ± 0.6 ***	62.5	125
*U. lactuca*	n.a	n.a	n.a
CuSO_4_	45.3 ± 0.6 ***	3.12	3.12
DMSO	n.a	n.a	n.a

Each value representing mean ± SD of six replicates, *** *p* < 0.001 indicates significant differences compared with DMSO, n.a not active.

**Table 4:** Inhibitory rate of *Cystoseira tamariscifolia* EO on *Microcystis aeruginosa*.

Treatments	Inhibition Rate (%)					
	Time (Days)					
	1	2	3	4	5	6
MIC	68.0 ± 0.4 ***	87.6 ± 0.4 ***	90.2 ± 0.5 ***	95.4 ± 0.1 ***	96.16 ± 0.08 ***	97.85 ± 0.05 ***
MBC	74 ± 1 ***	89.9 ± 0.2 ***	94.4 ± 0.3 ***	97.8 ± 0.1 ***	98.81 ± 0.07 ***	99.24 ± 0.07 ***
CuSO_4_	71 ± 2 ***	88.9 ± 0.2 ***	94.12 ± 0.07 ***	97.54 ± 0.05 ***	98.5 ± 0.02 ***	98.87 ± 0.01 ***
DMSO	−0.3 ± 0.5	−0.08 ± 1.67	−0.15 ± 0.39	−1.0 ± 0.6	−1 ± 2	−1.1 ± 0.9

MIC: minimum inhibitory concentration of *C. tamariscifolia* EO (7.81 μg mL^−1^) and MBC: minimum bactericidal concentration of *C. tamariscifolia* EO (15.62 μg mL^−1^). Each value representing mean ± SD of three replicates, *** *p* < 0.001 indicate significant differences compared with DMSO.

**Table 5:** Date of harvesting and location of the Moroccan seaweeds studied.

Species	Species Code	Harvesting Place	Date of Harvesting	Latitude/Longitude
*C. tamariscifolia*	Ct	Souiria Laqdima	February 2019	N 32°03′04.6″/W 9°20′30.2″
*S. muticum*	Sm	El jadida	April 2019	N 3°15′45.9″/W 8°30′03.4″
*U. lactuca*	Ul	El jadida	March 2019	N 3°15′45.9″/W 8°30′03.4″
